# Routine Healthcare Facility– and Antenatal Care–Based Malaria Surveillance: Challenges and Opportunities

**DOI:** 10.4269/ajtmh.22-0182

**Published:** 2022-06-06

**Authors:** Julie R. Gutman, Julie Thwing, Julia Mwesigwa, Peter D. McElroy, Molly Robertson

**Affiliations:** ^1^ Malaria Branch, Division of Parasitic Diseases and Malaria, Centers for Disease Control and Prevention, Atlanta, Georgia;; ^2^ PATH, Kampala, Uganda;; ^3^ PATH, Seattle, Washington

## Abstract

Most monitoring and evaluation tools for measuring malaria burden, intervention coverage, and impact of interventions use periodic nationally representative cross-sectional household surveys. These provide advantages in terms of selecting a large, unbiased, population-based sample; however, they are infrequently conducted, are resource-intensive, and do not provide longitudinal data with sufficient granularity. Given the heterogeneity of malaria transmission within most endemic countries, systems with the capacity to provide more granular and frequent data would be more actionable by national malaria control programs and local implementing partners. There is increasing interest in using routine health facility data, usually from outpatient department visits, for monitoring malaria burden. Data from pregnant women attending antenatal care (ANC) could minimize bias related to fever care-seeking among outpatient department visits and provide more granular parasite prevalence data. Most pregnant women attend ANC at least once and are thus highly representative of the overall pregnant population. A growing body of evidence suggests that malaria parasitemia in pregnant women is correlated with parasitemia in children aged < 5 years in moderate to high transmission areas, allowing for monitoring parasitemia in real time. Additional data are needed to assess whether pregnant women are sufficiently representative of the overall population to yield valid malaria prevalence and intervention coverage estimates. Although use of routinely collected ANC data faces many of the same challenges experienced by other routinely collected health facility data, the opportunity to improve parasite prevalence monitoring and the associated health benefits to mothers and infants of early detection of parasitemia make these efforts valuable.

## BACKGROUND

Surveillance methodologies for malaria in moderate and high transmission settings have traditionally focused on two methods: cross-sectional community household surveys to assess malaria prevalence^1^ and aggregate data of malaria cases from routine outpatient visits at health facilities to assess malaria incidence.^2^ This article details the benefits and challenges of a third methodology of estimating malaria parasite prevalence: using pregnant women as a sentinel population through routine antenatal care (ANC) visits. HIV programs have used pregnant women as a sentinel population for many years.^3^

There is a strong foundation and rationale for both of the traditional methods for estimating malaria burden. Cross-sectional community surveys provide detailed, population-representative information about malaria prevalence that is not influenced by care-seeking behavior or access to health services. They provide information about both symptomatic and asymptomatic malaria and, through a standard interview component—in most cases, the Malaria Indicator Survey format—this methodology can provide information about health-seeking behavior, health access, and access to and use of malaria preventive and curative interventions. These surveys can also be scaled to different administrative levels, depending on available resources. Budget and time constraints are among the key drawbacks of cross-sectional surveys. Powering the surveys to estimate malaria prevalence at even a regional level can incur substantial costs, and powering the surveys to a more useful health district operational level would be cost-prohibitive. With limited monitoring and evaluation budgets, this can put a strain on the resources for implementation. Due to cost and time factors, cross-sectional surveys typically provide intervention coverage and prevalence estimates limited to a provincial or regional level and are conducted only intermittently, generally not more than every 2 to 3 or more years.^4^ In addition, it usually takes several months after the survey for data to be entered, cleaned, analyzed, and disseminated. This puts obvious constraints on the utility of these data for timely and subnationally stratified programmatic decision-making.

Use of routine malaria case data from outpatient health facility visits has different benefits and drawbacks. These data provide information at a more granular (facility) level. Routine data are also aggregated monthly (sometime weekly), allowing assessment of spatial and temporal trends in both case incidence and test positivity rates. However, several major factors constrain the use of these data. First, accurate incidence calculations require an accurate population denominator for the administrative unit (e.g., health facility catchment area) used for analysis. The smallest unit, the health facility, requires knowledge of the catchment area around the health facility to assess how many at-risk persons reside at that location. Although these data exist, they are often not of high quality or updated routinely. In addition, these data often conflict with other administrative sources of population data, including summary reports from intervention assessments (such as household counts for insecticide-treated bed-net campaigns) and official government statistics, making it difficult to determine what population should ideally be used for the basic calculations of malaria cases per population.

Most critically, care-seeking for symptomatic cases is still low in much of sub-Saharan Africa and, in particular, may be affected by many equity issues, including distance to health facility, socioeconomic status, and the costs of healthcare.^5^^–^^8^ Both distance and socioeconomic status are also associated with a greater likelihood of malaria.^9^^–^^13^ Even when care is sought, it may not be at service providers that use rapid diagnostic tests (RDTs) or microscopy and report their data. Some attempt to address these challenges can be made by interpreting routine case data in light of care-seeking behavior from the most recent cross-sectional survey.^14^ In addition to care-seeking behavior, diagnosis at the health facility relies on multiple circumstances confronting the provider needing to perform a diagnostic test, including availability of the test, ability to detect febrile illness, and clinical assessment that the patient is a suspect malaria case. Ultimate inclusion of the malaria case in routine data systems also requires the provider to record and report it. Finally, by definition outpatient department data sample only a symptomatic population, which rules out understanding the contribution of asymptomatic infections when tracking routine health facility data.

The third possible methodology, malaria surveillance at ANC visits, overcomes many of these challenges. First, ANC attendance throughout malaria endemic areas is usually high; the 2021 World Malaria Report estimates that among the 33 countries with data on intermittent preventive treatment in pregnancy, 74% of women attended ANC at least once during their pregnancy.^14^ The reason for ANC visits is generally unrelated to malaria infection status, so these women’s malaria infection status could be considered a valid reflection of transmission levels in the communities where they live. Several studies have demonstrated that prevalence among pregnant women, particularly those in their first pregnancy (primigravidae), is highly correlated with prevalence among children under age 5 years obtained from household surveys.^15^^–^^17^

## BENEFITS OF ANC SURVEILLANCE

Through an ANC surveillance strategy, we could infer population malaria prevalence down to the health facility level every month. Like routine malaria case data recorded at outpatient department visits, women come to ANC visits throughout the month, and their information is recorded and summarized in routine data systems.^4^^,^^16^ The critical difference, however, is that ANC visits are likely to be among both asymptomatic and symptomatic persons, and outpatient department visits are likely to be among symptomatic persons only. The ANC data allow us to observe and analyze more generalizable trends corresponding to seasonality, the introduction of malaria interventions, and the emergence of outbreaks. These data also allow for stratification of malaria burden at a health-facility level, which could aid decision-making in a resource-constrained environment.

For the analysis phase, ANC surveillance presents two key benefits. First, given that women are seeking care for a purpose other than febrile illness, the prevalence of asymptomatic malaria can be assessed in a way that is not possible through outpatient data, where the number of people being tested is dependent not only on symptomatic cases but also on the prevalence of any other febrile illnesses circulating at that time. In addition, the denominator for analysis of prevalence does not rely on reference to a difficult-to-determine catchment population. Instead, the population that comes in for a first ANC visit becomes the denominator. It is then a calculation of the number of women who tested positive for malaria (usually by RDT) in their first ANC visit divided by the total number of women who attended that month (or even that week or day) for their first ANC visit. Although there are limitations, the possibility that ANC surveillance can be granular, can be monthly, can give us information about asymptomatic cases, and can be easily calculated, makes it crucial to consider this method for monitoring malaria parasite prevalence in the community.

Although less pertinent for surveillance, it must be noted that there is also a potential benefit for the pregnant women and fetus of screening for malaria at the time of first ANC visit. Malaria infection in early pregnancy can result in maternal anemia, fetal loss, premature delivery, intrauterine growth retardation, and low birth weight.^18^ Currently, the only malaria control measure available for prevention among pregnant women in first trimester is insecticide-treated net use, which does nothing to address women who are already infected. By testing each pregnant woman for malaria infection at first ANC visit and treating confirmed cases, the risk of adverse pregnancy outcomes can be diminished; this is particularly true for women who present for their first ANC visit in the first trimester.^4^^,^^19^^,^^20^

In this article, we primarily discuss ANC surveillance as a methodology for estimating malaria burden, but it may also be possible to analyze other key indicators typically assessed through cross-sectional survey questionnaires. These include access to and use of other interventions, such as insecticide-treated nets, health-seeking behavior for the woman and for her children, and economic expenditures related to malaria. As with prevalence data, the addition of these indicators comes with additional challenges.

## CHALLENGES OF ANC SURVEILLANCE

ANC surveillance can be an important tool in estimating malaria infection prevalence and informing malaria decision-making, but it is not without its challenges. These challenges are important to document and assess. A primary challenge is the ability of the nurses responsible for a range of ANC services to take on the additional malaria testing and treatment burden. At its simplest level, this means adding a malaria test (most likely RDT) to the current battery of tests that often accompany a first ANC visit; this can be incorporated alongside routine testing for anemia, syphilis, and HIV, without requiring an additional finger prick or venous blood draw. This requires minimal explanation because women living in malaria endemic areas are aware of malaria and the risk associated with it and welcome the opportunity to be tested for malaria.^21^ Nurses are also well versed in malaria RDT procedures and, in accordance with most case management procedures, will already perform an RDT for any women presenting or reporting malaria symptoms during these visits, and treat anyone testing positive. Therefore, conducting RDTs for all women attending their first ANC visit does not introduce substantial additional time or burden^21^ and, at approximately USD $0.35 per RDT, is relatively inexpensive.^22^

This changes, however, if other malaria programmatic indicators are introduced. Asking each patient for detailed information on household insecticide-treated net ownership and use, and care-seeking history for her children can take a substantial amount of time. Each additional question must be weighed carefully to assess the benefits against the increased time for the nurse and patient. Additional questions added to the ANC register will be subject to the same challenges with accurate data aggregation and upward reporting normally seen with any other routinely reported indicators. Challenges of data recording and reporting, and associated issues of data quality, take on an outsized burden. Finally, implementing such a system requires cooperation and coordination among a country’s national malaria control department, maternal health department, and health management information system (HMIS) department.

Women have been shown to develop some level of acquired immunity through the pregnancy period that manifests further with each subsequent pregnancy.^18^ Therefore, women who have already carried one or multiple children may be less representative of the overall community population than those women who are pregnant for the first time, but additional data are needed to better assess this issue. This can be accounted for by categorizing and calculating prevalence among women in their first pregnancy (primigravid), second pregnancy (secundigravid), or third or more pregnancy (multigravid). Information on the number of previous pregnancies is asked at every first ANC visit and recorded in the ANC register. However, this information exists solely at the individual record level. When aggregating data monthly, as is done for upward reporting of data to the central HMIS level, in which data are entered into the District Health Information Software, version 2 (DHIS2), this information is lost because data elements are summarized by column rather than entered individually by row. For example, Maria, age 22, on her second pregnancy and malaria positive, becomes aggregated with other women to reflect average age (Maria who is 22 plus Ines who is 30 and Anna who is 28), and gravidity is also summarized (40% were primigravid, 30% were secundigravida). This data aggregation also happens with the RDT results, so it would be reported that one out of three women was malaria-positive, but we would not know whether that one woman was primigravid.

With enough data, it might be possible to estimate the likely community burden with these aggregates, but currently there are not enough analyzed data to make the correlation. If women in their first and second pregnancies are shown to be most relevant for surveillance, one option might be to perform testing only on these women, although that would introduce some logistical challenges for clinics in terms of ensuring that the correct women were tested. In addition, it would limit the number of women providing data from individual clinics such that data would need to be aggregated either over more time or across facilities to provide reliable estimates. Alternatively, countries could consider investing in newer digital technologies, such as digital image recognition software, which would allow individual patient data collected in paper registers to be captured by digital photography and uploaded directly to the cloud (e.g., ScanForm, https://about.scanform.qed.ai/). While collecting individual-level data digitally would provide substantial benefits in terms of analytic possibilities, it also has privacy implications that must be addressed to ensure adequate protection of patient information.

To ultimately render ANC surveillance data most useful requires patient-specific records. Currently, collecting individual-level data is not part of most routine ANC data collection procedures implemented by HMIS. As with the addition of indicators on care-seeking behavior and intervention use, a parallel data collection method would need to be used. This could be paper-based or electronic but would require the reentering of all the relevant indicators from the ANC register. Whether register summaries or reentering of register data is used, the challenges of routine data, including data quality, completeness, and validity, are just as much of a challenge with ANC surveillance as with other routine data. The use of digital registers for ANC is also subject to the same limitations as digital registers in outpatient services—namely, lack of electricity, connectivity, the expense of procuring and upkeep of tablets or phones, and the need for continual supervision and refresher trainings as staff turnover.^23^^,^^24^ Adding to this complication is the often separate departments, supervision, and management of ANC staff, staff responsible for malaria in outpatient services, and staff for data reporting.

Even if most women attend at least one ANC visit, if a large proportion of women attend ANC at private facilities, which do not report into the National HMIS, this will affect data quality, particularly as the populations selecting private facilities may be very different from those at public facilities in terms of malaria risk. Nonetheless, if this system is looked at as a way to assess response to interventions and identify hot spots, it may still have some value. Ideally, in countries where utilization of private health facilities is high, those facilities would submit data to the HMIS. Additionally, while overall ANC utilization is generally high, some countries, and specific areas within countries, may have lower ANC attendance, which would affect the generalizability of the data. As noted earlier, equity issues such as farther distance to health facility and lower socioeconomic status decrease the likelihood of seeking ANC as well as increase the risk of malaria.^5^^–^^13^

Another challenge, one not to be underestimated, is the necessary attention to stock procurement and distribution planning/execution to ensure health facilities have sufficient RDTs for every woman coming for a first ANC visit and antimalarial treatment of those who test positive. The addition of a small number of RDTs may seem minor, but it requires an adjustment in procurements and supply chains, calculations between different departments, levels of permission to sign off on additional stocks leaving provincial and district warehouses, and reliable transportation to ensure that the stocks arrive routinely. ANC surveillance should never compromise the stocks needed for diagnosis and treatment of symptomatic malaria and, in cases in which the stocks are low, the health facility would rightly revert to testing only pregnant women with malaria symptoms; however, that would adversely affect the validity of the surveillance data and require adjusting the data if this occurred.

## CONCLUSION

Although there are many challenges with ANC-based malaria surveillance, as there are with all other forms of surveillance data, the possibilities of enhancing our understanding of malaria burden through ANC surveillance are clear. As we move forward with strengthening outpatient malaria surveillance, building digital health infrastructure, and fortifying data analysis and decision-making for stratification, the malaria control community should consider the ANC platform as a strategy to complement existing facility based malaria surveillance. There has been a considerable effort over the past few years to build a robust evidence base to support that ANC surveillance can be used to predict the patterns of malaria prevalence among children under age 5, although further analysis is needed to refine the models and better describe the relationship between community malaria prevalence and ANC prevalence. In the meanwhile, there are sufficient data to show that ANC surveillance is more sensitive at detecting low levels of ongoing transmission than demographic health surveys, and countries should consider how this fits into their overall surveillance package.
